# Nutrients and Oxidative Stress: Friend or Foe?

**DOI:** 10.1155/2018/9719584

**Published:** 2018-01-31

**Authors:** Bee Ling Tan, Mohd Esa Norhaizan, Winnie-Pui-Pui Liew

**Affiliations:** ^1^Department of Nutrition and Dietetics, Faculty of Medicine and Health Sciences, Universiti Putra Malaysia, 43400 Serdang, Selangor, Malaysia; ^2^Laboratory of Molecular Biomedicine, Institute of Bioscience, Universiti Putra Malaysia, 43400 Serdang, Selangor, Malaysia; ^3^Research Centre of Excellent, Nutrition and Non-Communicable Diseases (NNCD), Faculty of Medicine and Health Sciences, Universiti Putra Malaysia, 43400 Serdang, Selangor, Malaysia

## Abstract

There are different types of nutritionally mediated oxidative stress sources that trigger inflammation. Much information indicates that high intakes of macronutrients can promote oxidative stress and subsequently contribute to inflammation via nuclear factor-kappa B- (NF-*κ*B-) mediated cell signaling pathways. Dietary carbohydrates, animal-based proteins, and fats are important to highlight here because they may contribute to the long-term consequences of nutritionally mediated inflammation. Oxidative stress is a central player of metabolic ailments associated with high-carbohydrate and animal-based protein diets and excessive fat consumption. Obesity has become an epidemic and represents the major risk factor for several chronic diseases, including diabetes, cardiovascular disease (CVD), and cancer. However, the molecular mechanisms of nutritionally mediated oxidative stress are complex and poorly understood. Therefore, this review aimed to explore how dietary choices exacerbate or dampen the oxidative stress and inflammation. We also discussed the implications of oxidative stress in the adipocyte and glucose metabolism and obesity-associated noncommunicable diseases (NCDs). Taken together, a better understanding of the role of oxidative stress in obesity and the development of obesity-related NCDs would provide a useful approach. This is because oxidative stress can be mediated by both extrinsic and intrinsic factors, hence providing a plausible means for the prevention of metabolic disorders.

## 1. Introduction

There are different types of nutritionally mediated oxidative stress sources that trigger inflammation. Oxidative stress plays a crucial role in the development of numerous human diseases [1]. Reactive oxygen species (ROS) and reactive nitrogen species (RNS) are produced continuously in the body via oxidative metabolism, mitochondrial bioenergetics, and immune function [2]. The most frequent forms of ROS include superoxide anion, hyphochlorous acid, hydrogen peroxide, singlet oxygen, hypochlorite, hydroxyl radical, and lipid peroxides, which are involved in the progression, growth, death, and differentiation of cells. They can bind with nucleic acids, enzymes, membrane lipids, proteins, and other small molecules [1]. Short-term postprandial mitochondrial oxidative stress causes inflammation, which is mainly mediated by nuclear factor-kappa B (NF-*κ*B) [3]. Conversely, long-term chronic overconsumption contributes to obesity, which induces permanent states of inflammation via the generation of white adipose tissue which secretes proinflammatory factors [4]. Extensive research has shown that high-glucose and a high-fat diet mediate inflammation, which suggests that oxidative stress may alter cellular physiological processes [5, 6].

Substantial evidence highlights the detrimental impact of diets high in refined carbohydrates and saturated fat [7]. Cardiovascular disease (CVD), obesity, type 2 diabetes, and nonalcoholic fatty liver disease are attributed to the overconsumption of foods high in carbohydrates and saturated fats, the saturation of nutrient storage, and sedentary lifestyles [8, 9]. Studies exploring the influence of a Westernized dietary pattern on inflammatory diseases, such as colorectal cancer [10], have consistently shown a similar trend. Such findings highlight the fundamental idea that diet quality can impact immune function and systematic inflammation. In a study by Song et al. [11] focusing on carbohydrate and refined-grain intake and metabolic syndrome outcome in Korean men and women, women were shown to have a greater likelihood of metabolic syndrome with refined-grain consumption compared to the men, suggesting that refined-grain intakes are linked to a high level of inflammation.

The prevalence of obesity has doubled from 1980 to 2008 worldwide. In 2008, more than 50% of men and women in the WHO European Region were overweight, and nearly 20% of men and 23% of women were obese [12]. Nearly 1.5 billion people worldwide are obese or overweight which increases their risk of developing inflammatory disturbances, CVD, nonalcoholic fatty liver disease, coronary heart disease, and type 2 diabetes [13, 14].

The effects of oxidative stress are related to the type of macronutrients consumed and their absolute quantity [15]; both of these aspects contribute to oxidative stress and may favor the development of obesity and obesity-related noncommunicable diseases (NCDs) [16]. However, the molecular mechanisms of nutritionally mediated oxidative stress are complex and poorly understood. Therefore, this review aimed to explore how dietary choices exacerbate or dampen oxidative stress and inflammation. We also discussed the implications of oxidative stress in the adipocyte and glucose metabolism and obesity-associated NCDs. A better understanding of the role of oxidative stress in obesity and the development of obesity-related NCDs would provide a useful approach. This is because oxidative stress can be mediated by both extrinsic and intrinsic factors, hence providing a plausible means for the prevention of metabolic disorders.

## 2. Oxidative Stress

The harmful effects of free RNS and ROS radicals cause a potential biological damage, namely, nitrosative stress and oxidative stress, respectively [17]. ROS are generated in normal aerobic metabolism as a by-product; however, when the level is increased under stress, it may cause basic health hazard [18]. The mitochondrion is the predominant cell organelle in ROS production [19]. It generates adenosine triphosphate (ATP) via a series of oxidative phosphorylation processes [19]. During this process, one or two electron reductions instead of four electron reductions of oxygen have occurred, which subsequently leads to the formation of H_2_O_2_ or O_2_^∙^, and convert to other ROS [19]. The major form of RNS includes nitric oxide (NO) and peroxynitrite (ONOO−) [17]. When excess NO is present, this reaction leads to the formation of nitrogen dioxide radical [17]. Higher NO concentration leads to the formation of N_2_O_3_ and this usually results in nitrosation [17].

Oxygen free radicals, including alkyl peroxyl radical (^∙^OOCR), hydroxyl radical (OH^∙−^), and superoxide anion radical (O_2_^∙−^), are potent initiators in lipid peroxidation, the role of which is well-established in the pathogenesis of diseases [18]. Once lipid peroxidation is initiated, a propagation of chain reactions will take place until termination products are produced [18]. Thus, end products of lipid peroxidation, for example, F2-isoprostanes, 4-hydroxy-2-nonenol (4-HNE), and malondialdehyde (MDA), are accumulated in biological systems [18]. DNA bases are very susceptible to ROS oxidation, and the major detectable oxidation product of DNA bases is 8-hydroxy-2-deoxyguanosine [18]. Oxidation of DNA bases can cause mutations and deletions in both nuclear and mitochondrial DNA. Mitochondrial DNA is relatively prone to oxidative damage due to its proximity to ROS and its deficient repair capacity compared to that of the nuclear DNA [18]. These oxidative modifications cause functional changes in structural and enzymatic proteins, which may lead to substantial physiological impact [18]. In addition, redox modulations of transcription factors also increase or decrease their specific DNA binding activities and thereby altering gene expression [18].

## 3. Nutritionally Mediated Oxidative Stress

### 3.1. High Carbohydrates

Much information indicates that high intakes of macronutrients can promote oxidative stress and subsequently contribute to inflammation via NF-*κ*B-mediated cell signaling pathways [20]. Dietary carbohydrates are important to highlight here because they may contribute to the long-term consequences of nutritionally mediated inflammation [21]. Dietary carbohydrate intake has gained attention among researchers because of the associations between a high glycemic index (GI) or glycemic load (GL) diet with diabetes, obesity, cancer, and coronary heart disease [22, 23]. High GL diets have been characterized as a common feature of Western culture; they are heavy in added sugars and refined carbohydrates [24]. By contrast, low GI foods were found to decrease postprandial glycemia in overweight/obese [23] and type 2 diabetes patients [25]. Consistent relationships between high GI and diabetes have been demonstrated in observational and cohort studies [26–28].

The high GI of white rice may lead to high oxidative stress [29]. Most Asian populations consume large amounts of rice as a staple food; thus, dietary carbohydrate intake plays a substantial role in the development of metabolic diseases in Asian populations. In support of this, a positive relationship between rice intake or total carbohydrates and diabetes has been demonstrated in Japanese women [30, 31]. In addition to diabetes outcome, a high intake of refined-grain was also positively linked to fasting blood glucose and triglyceride levels and negatively associated with high-density lipoprotein (HDL) cholesterol in Asian Indian and Korean populations [11, 32], indicating that a high GI diet may negatively impact health.

The elevation of oxidative stress is linked to chronic inflammation [33]; other sources may also further increase the accumulation of proinflammatory cytokines in a “vicious cycle” [34]. In cultured adipocytes, ROS promotes the production of cytokine interleukin-6 (IL-6) and proinflammatory monocyte chemotactic protein-1 (MCP-1) expression [35, 36]. In the adipose tissue, this can activate macrophage infiltration and subsequently result in a proinflammatory environment [37, 38]. ROS can also stimulate signal transduction pathways (mainly via NF-*κ*B), which activates the production of tumor necrosis factor-*α* (TNF-*α*) and IL-6 [35, 39, 40]. Further, oxidative stress can also promote cells into cellular senescence, particularly adipocyte senescence, partly via cellular oxidation damage [41, 42]. Adipocyte senescence may recruit macrophages and elevates the production of proinflammatory cytokines [42, 43].

Excessive high caloric intake from either a high-carbohydrate or high-fat diet will cause more substrates to enter into mitochondrial respiration [44]. Subsequently, the number of electrons donated to the electron transport chain may increase [45]. Upon reaching a threshold voltage, extra electrons might back up at complex III with further donations to molecular oxygen, which produces high levels of superoxide [45].

Intriguingly, extremely high amounts of carbohydrates may lead to the reduction of insulin binding and the downregulated transcription of insulin receptor expression in the skeletal muscle [46]. High insulin and glucose levels may decrease insulin binding to the insulin receptor in adipocytes [47], negatively affecting Akt activity. The accumulation of ROS/RNS or a reduction of antioxidant capacity due to increased carbohydrate metabolism in insulin target tissues may change the phosphorylation status of these signaling pathways, subsequently resulting in deactivation. Indeed, exposure to hydrogen peroxide (H_2_O_2_) promotes a significant loss in distal and proximal insulin signaling and decreased glucose transport in muscles and adipocytes *in vitro* [48].

Evidence from an epidemiological study demonstrated that the consumption of refined carbohydrates, such as fructose-rich syrups, potentially leads to the epidemics of type 2 diabetes and obesity [49, 50]. Indeed, fructose-rich syrups may potentially pose a risk of diabetes and CVD [49]. Animal model studies further demonstrate that feeding normal rats fructose-rich diets may induce several endocrine and metabolic derangements, interfering with many organs and tissues [51, 52]. Because the liver is predominantly responsible for fructose metabolism and uptake, several studies are focusing on hepatic glucose metabolism [53]. Although the molecular link underlying fructose detrimental effects and carbohydrate metabolism requires further elucidation, most of the experimental studies indicate that oxidative stress could play a central role [54, 55]. In this regard, a key mode of action to explain this relationship is via fructose-induced oxidative stress which subsequently leads to impaired carbohydrate metabolism. Data from animal experiments have shown a greater likelihood of inflammation after the administration of fructose [51]. Such findings highlight the association of insulin resistance and fructose and its role in hepatic metabolism and carbohydrate metabolism against the anabolic pathway and impaired glucose tolerance [52, 55, 56]. Castro et al. [53] further demonstrated that fructose may modulate the liver glucokinase activity via the production of ROS. These data imply that numerous metabolic changes induced by fructose in the liver are more likely initiated by an increase of fructose phosphorylation by fructokinase, followed by adaptive changes that attempt to switch the substrate flow from mitochondrial metabolism to energy storage [53].

### 3.2. High Animal-Based Proteins

In developed countries, meat composes a significant proportion of the normal diet and consists of 15% of the daily energy intake, 40% of daily protein, and 20% of daily fat [57]. Meat is high in dietary protein and saturated fatty acids (SFAs). Fermentation of the excessive proteins in the gut produces metabolites such as ammonia (NH3) and hydrogen sulfide (H2S), which are compounds known to trigger the toxicity of the mucosa [58]. Meat can be marketed fresh or processed, the latter of which includes curing, salting, stuffing, smoking, drying, and fermentation [59]. Although meat contains high amounts of dietary protein, it can also be a source of mutagens due to the presence of N-nitroso compounds (NOC) in processed meats and heterocyclic amines (HCA) and polycyclic aromatic hydrocarbons (PAH) during high-temperature cooking and grilling [60].

Research has shown an association between the intake of well-done red meat and colorectal cancer, which could be partially explained by the formation of carcinogenic HCA and PAH. Although meat is high in SFAs, a study evaluating the mechanisms behind this finding suggests that these associations are more likely caused by something other than SFA content. However, the formation of cyto and genotoxic lipid oxidation products, such as malondialdehyde (MDA), 4-hydroxy-2- nonenal (4-HNE), and N-nitroso compounds (NOC) catalyzed by heme-Fe during digestion, is regarded as the most plausible determinant that contributes to the increased risk of colorectal cancer [61, 62]. A high intake of red meat has been demonstrated to increase NOC formation in humans, which is related to the colonic development of the NOC-specific DNA adduct O6-carboxymethylguanine (O6-C-MeG) [63].

Free Fe^2+^ markedly increases during the cooking of uncured meats [63]. Conversely, nitrite curing prevents the degradation of heme-Fe through the stabilization of the porphyrin ring [63]. Heat treatment also causes a reduction of antioxidant enzymes, such as glutathione peroxidase [64, 65], and generates oxygen from oxymyoglobin, which contributes to the production of H_2_O_2_ [66]. Further, free Fe^2+^ catalyzes the Fenton reaction when oxidative processes are initiated [67]. Through this reactive nature, ROS results in oxidative damage to meat proteins, which further explains the high formation of 4-HNE and MDA when uncured pork is heated [68]. Compared to cooked meat, a slightly lower concentration of simple aldehydes was observed in overcooked uncured pork. This could be explained by the evaporation of aldehydes caused by the reduction of the prooxidant effect of oxymyoglobin when heated to above 75 °C or intense heating [69]. Rather, when meats are nitrite-cured, less degradation of the heat-stable NO-heme may contribute to a reduced release of Fe^2+^ to initiate oxidation processes, which subsequently results in a reduction of lipid oxidation. Because the Fenton reaction is a chain reaction, a higher dosage of oxidation products after digestion was expected [70]. A further study reported by Van Hecke et al. [63] showed that the antioxidant effect of nitrite-curing during digestion was significantly reduced in overcooked nitrite-cured pork. Consistent with the study reported by Van Hecke et al. [63], Okayama et al. [71] also found that a prolonged cooking time or a temperature reaching 80 °C increased the decomposition of nitrite. A 1 : 1 ratio of nitric oxide (^∙^NO) to ROS activates lipid oxidation whereas ^∙^NO > ROS suppresses this process [72]. Accordingly, low residual nitrite caused by intense heating is more likely to alter the ^∙^NO : ROS ratio; thus, nitrite could change from an antioxidant to prooxidant behavior, which might explain the increased formation of oxidation products in overcooked nitrite-cured meats. In an earlier study by Ayala et al. [73], MDA was shown to be absorbed in the bloodstream and produce lipid oxidation products that could reach tissues and cause DNA damage. Low lipid oxidation product levels in colonic digests are attributed to Schiff base formation with proteins, which thus binds with bacterial DNA [74] or is oxidized by bacterial aldehyde dehydrogenase activity. Collectively, the effect of nitrite curing of meat in the colonic step was predominant since it was linked to a low level of MDA but proportionally increased 4-HNE levels and doubled heptanal amounts in the overcooked and cooked meats [63].

In addition, the nitrite-curing of beef and pork also caused a twofold difference in heptanal levels in stimulated colonic digests compared to their counterparts [70]. Lipid aldehydes, such as 4-HNE and MDA, react with protein chains leading to protein aggregation, causing the protein to be less susceptible to pepsin activity [70]. Overcooked nitrite-cured pork has low concentrations of protein carbonyl compounds and lipid oxidation products before digestion; this likely occurs because the meat proteins are initially well-digested in the stomach, after which the low levels of residual protein bind with 4-HNE and MDA, which subsequently form in a later phase of digestion [70]. The rate of protein digestibility is vitally important in association to colorectal cancer because higher levels of residual protein reaching the colon could result in the formation of potentially harmful protein fermentation products, such as p-cresol, ammonia, indole, and phenol [75]. NOC can be stimulated either enzymatically or nonenzymatically via oxidation [76, 77]. This nonenzymatic stimulation of NOC can be generated by a hydroxyl radical-generating system containing H_2_O_2_, Cu^2+^, Fe^2+^, and ascorbic acid. All these compounds are present in meat. When H_2_O_2_, Cu^2+^, or Fe^2+^ was eliminated from a reaction mixture with N-nitroso-N-methylpentylamine, the mutagenicity of these mixtures was reduced [76].

### 3.3. Excessive Consumption of Fats

An extensive body of systematic reviews of randomized trials [78, 79] and prospective cohort studies [78, 80] has urged for a reevaluation of dietary guidelines for consumption and a reappraisal of the impact of SFAs on health. Although research has demonstrated an association between SFAs and CVD [81], not all data demonstrated such a link. De Souza et al. [82] did not identify an association of SFA intake and CVD, coronary heart disease, ischemic stroke, or type 2 diabetes. Interestingly, a study has reported that the total fat and types of fat were inversely associated with total mortality [83]. Additionally, no association was reported between the total fat and types of fat with CVD mortality and myocardial infarction [83].

Substantial evidence has suggested that SFAs can boost proinflammatory signaling. The lengths of SFA chains can produce different physiological responses [84, 85], but many mechanisms are still debated. Long-chain SFAs including palmitate and myristate acids are typically known for their harmful effects against endothelial cells, which can induce apoptosis through the induction of NF-*κ*B in human coronary artery endothelial cells (HCAECs) [86, 87]. Harvey et al. [86] showed that long-chain SFAs can promote proinflammatory endothelial cell phenotypes through the incorporation into endothelial cell lipids. Conversely, short- and medium-chain SFAs do not incorporate or contribute to lipotoxicity. Particularly, stearic acid stimulates the upregulation of *ICAM-1* human aortic endothelial cells (HAECs) via an NF-*κ*B dependent manner [86].

Murumalla and Gunasekaran [88] reported that SFAs (lauric acid and palmitic acid) did not stimulate Toll-like receptors 4 (TLR4) or 2 (TLR2) in HEK-Blue cells transfected with TLR2 and TLR4. Despite the inverse association between SFAs and TLR4 or TLR2, not all studies agreed. Huang et al. [84] found that palmitic acid and lauric acid activated TLR2 and TLR4 in RAW264.7 macrophages and transiently transfected human monocytic (THP-1) monocytes. Data from human studies exploring the impact of SFAs on gene expression are limited, but evidence from epidemiological studies indicates the association between SFA consumption and CVD. Nonetheless, the meta-analyses of prospective studies exploring the relationship between CVD and SFA showed a consistent poor association. From the study reviewed, metaregressions conducted in randomized trials demonstrated that polyunsaturated fatty acids (PUFAs) replacing SFAs did not lead to any changes in CVD risk [89]. Inconclusive findings suggest that SFAs are generally grouped together although medium-chain SFAs may provide beneficial health effects such as preventing obesity and the inhibition of body fat accumulation [90]. The impact of high-SFA diet on gene expression in adipose tissue was also presented by Youseef-Elabd et al. [91]. In particular, an SFA diet led to an upregulation of genes such as integrin beta 2 (*ITGB2*), cathepsin S (*CTSS*), and interleukin-8 (*IL-8*) in moderately overweight individuals, suggesting that these changes were linked to diet-induced changes rather than obesity.

A high-fat diet (HFD) was demonstrated to be a significant risk factor for health. Animals feeding a long-term HFD show increased oxidative stress and dysfunctional mitochondria in several organs [92–94]. Several research studies have also indicated that high-fat consumption causes a significant reduction in auditory function [95, 96]. This study demonstrated that long-term HFD reduced auditory function and promoted age-related hearing loss [97]. From the study reviewed, feeding rats with a HFD for a period of 12 months may increase plasma triglycerides, total cholesterol, and nonesterified fatty acid levels, causing an increase in blood oxidative stress parameters. A HFD was shown to not only aggravate the lipid profile it also further enhanced ROS accumulation and triggered mitochondrial damage in the inner ear [97], suggesting enormous detrimental impacts of a HFD on health.

Several studies have corroborated this finding and found that increased caloric intake or obesity is associated with increased mitochondrial superoxide production. Data reported by Anderson et al. [98] have shown that feeding a HFD to both mice and humans causes a significant elevation of H_2_O_2_ from the mitochondria isolated from the skeletal muscle. From the study reviewed, H_2_O_2_ emission was used as a surrogate of superoxide emission as mitochondrial superoxide and is converted to H_2_O_2_ by superoxide dismutase 2 (SOD 2). Further, ROS accumulation has also been found in mitochondria isolated from adipose [99], liver [100], and kidney [101] tissue in high-fat or obese-treated animals. In another study, Valenzuela et al. [102] found that liver enzyme activity such as superoxide dismutase (SOD), catalase, glutathione peroxidase, and glutathione reductase was significantly reduced by a HFD diet-fed mice.

Additionally, an adipogenic diet and the accumulation of adipose tissue can trigger oxidative stress in mammalian tissues. Some studies supported the hypothesis that HFD promotes inflammation in the intestine, particularly in the small intestine. This observation may represent an early event that precedes and predisposes the individual to insulin resistance and obesity [103]. de La Serre et al. [104] reported that HFD activates myeloperoxidase activity, an inflammation marker, in the ileum of obesity-prone Sprague-Dawley rats. A study by de Wit et al. [105] further supported that HFD activates macrophage migration inhibitory factor expression in the ileum of obesity-prone C57BL/6J mice. Consistent with studies reported by de La Serre et al. [104] and de Wit et al. [105], Ding et al. [106] and Cortez et al. [107] also found that TNF-*α* expression was activated after 2 to 6 weeks of HFD administration and led to weight gain and an increased body fat mass. High-fat consumption also stimulates Kupffer cells (the resident macrophages of the liver) in mice and causes an elevation of the M1-polarized population, which is linked to the pathogenesis of obesity-induced fatty liver disease and insulin resistance [108]. Consequently, obesity is associated with a marked increase in oxidative damage to all cellular macromolecules [14, 109, 110].

The mechanisms underlying the elevation of oxidative stress in metabolic disorders are not fully understood, but it is hypothesized that mitochondrial dysfunction [16], augmented by NADPH oxidase activity [111], and increased fatty acid oxidation [112] contribute to these phenomena. Most of the studies so far addressed abnormal gene expression in the adipose tissues and liver, accompanied by upregulated NADPH oxidase expression and downregulated antioxidative enzyme expression [113, 114]. HFD promotes dyslipidemia, which is associated with oxidative stress, an accumulation of some transition metals and elevated free radicals [115]. Fat accumulation has also been linked to systemic oxidative stress in mice and humans via the increased accumulation of ROS, accompanied by the improved expression of NADPH oxidase and the decreased expression of antioxidative enzymes [114]. Moreover, HFD provokes lipid peroxidation and oxidative stress, whereas NADPH oxidase activation deregulates the production of redox-sensitive transcription mRNA such as NF-*κ*B and adipocytokines (fat-derived hormones) including plasminogen activator inhibitor-1, monocyte chemotactic protein-1 (MCP-1), IL-6, adiponectin, and other inflammatory cytokines form different metabolic tissues [116].

Additionally, HFD raises the level of chylomicrons in the intestine. These chylomicrons enter circulation and cause the generation of free fatty acids (FFAs), which are taken up by the liver. These hepatic FFAs may either enter the mitochondria for *β*-oxidation or be esterified into triglycerides [117, 118]. Triglycerides are either accumulated in hepatocytes as small droplets or generate very low-density lipoprotein (VLDL) which is thereby converted into low-density lipoprotein (LDL) [118]. An excessive LDL burden in the blood due to its excessive accumulation or lack of LDL-receptors in hepatocytes may form oxidized-LDL (Ox-LDL), which in turn is engulfed by macrophages to become foam cells. Subsequently, foam cells accumulate in the arterial endothelium to form plaque. Ultimately, these lead to cardiovascular and circulatory disorders such as thromboembolism, hypertension, atherosclerosis, and heart block [119–121]. Subsequently, the mitochondrial *β*-oxidation of FFAs is linked to the conversion of oxidized cofactors (NAD^+^ and FAD) into reduced cofactors NADH and FADH_2_ and is thereby reoxidized and restored back into NAD^+^ and FAD by the mitochondrial respiratory chain. During reoxidation, NADH and FADH_2_ transfer electrons to the first complexes of the respiratory chain. Most of these electrons then migrate up to cytochrome-c oxidase and thereby combine with protons and oxygen to form water. These intermediates may interact with oxygen and produce more and more superoxide anion radicals and other ROS [122–125]. Therefore, the high consumption of fat-rich diets promotes mitochondrial *β*-oxidation of FFAs and subsequently leads to an excess electron flow using cytochrome-c oxidase, which elevates the accumulation of ROS. Mitochondria are a vitally important cellular source of ROS; they oxidize the unsaturated lipids of fat deposits to cause lipid peroxidation. ROS and lipid peroxidation can consume vitamins and antioxidant enzymes [125, 126]. The depletion of these protective substances may hamper ROS inactivation and promote ROS-mediated damage and lipid peroxidation [114]. This HFD-induced ROS may stimulate the proinflammatory state and thereby activate the NF-*κ*B transcription factor. Further, HFD also may trigger ROS or NF-*κ*B, which induces NF-*κ*B-dependent proinflammatory agents such as TNF-*α*, inducible nitric oxide synthase (iNOS), and interferon-*γ* (IFN-*γ*) [101, 127, 128]. These data converge to provide evidence supporting the role of oxidative stress induced by HFD in metabolic disorders. Surprisingly, an *in vitro* study showed that free fatty acids increased ROS accumulation, indicating that increased fatty acids in obesity may provide an extra source of additional electron transport chain substrates via the oxidation of fatty acids [111, 129]. In addition to the generation of ROS, the overproduction of nitric oxide (NO) through the activation of iNOS also causes an accumulation of RNS [130, 131]. Taken together, chronic consumption of high GI foods may cause oxidative stress via the formation of free radicals that are capable in destroying biological molecules and initiate abnormal cell growth through gene mutation [132]. Further, HCA formed during high-temperature cooking and grilling of meat may cause oxidation of proteins and lipids, thereby resulting in oxidative stress and may subsequently increase the risk of chronic diseases [133], while the HFD may serve as a stimulus to elevate the systemic inflammatory response in the development of obesity, CVD, diabetes, and cancers [134–137]. Overall, these data imply that high-carbohydrate/high-calorie/high-fat diets stimulate oxidative stress by augmenting the inflammatory response and elevating inflammatory markers.

## 4. Molecular Connectivity of Oxidative Stress-Induced Diseases

### 4.1. Obesity and Adipocyte Dysfunction

Obesity has been recognized as a heritable disorder in recent decades [138]. It has become increasingly clear that sedentary lifestyles and an increased availability of inexpensive calorie-dense foods have played a pivotal role in creating an obesogenic environment, which has contributed to the obesity epidemic [139–141]. The individual heritability of obesity susceptibility genes and interaction of the nutrients in the obesogenic environment, particularly dietary macronutrients, including refined carbohydrates and saturated fats, are linked to weight gain and may subsequently contribute to obesity [142, 143]. Thus, the functions of obesity susceptibility genes may be associated with this major health concern.

Obesity is considered a chronic low-grade inflammatory stress condition modulated by immune cells via the infiltration of adipose tissue, along with metabolic stress when oversupplied with glucose and lipids in adipocytes [144, 145]. Inflammatory cytokines have been observed in many fat cells; they are involved in fat metabolism and are associated with all indices of obesity, particularly abdominal obesity [146]. The alterations of leptin and hypothalamic pituitary adrenal (HPA) axis dysfunction, adipocyte function, and fatty acid levels and oxidative stress have been suggested to play a vitally important role in obesity-associated inflammation [146]. In general, the association between excessive nutrient uptake (sugars, lipids, and fatty acids) and metabolic disturbances is modulated by several types of cells, such as adipocytes and resident or infiltrating immune cells including monocytes, T cells, mast cells, and macrophages, which indirectly modify adipocyte function and dysfunction [147, 148]. A study by Lim et al. [149] found that dietary fatty acids activate protease-activated receptor 2 (PAR2) expression, which is a new biomarker for obesity and a substantial contributor in metabolic dysfunction and inflammation.

Studies have shown that ROS is generated from hypertrophic adipocytes induced by a HFD. The expansion of fat mass occurs via two concomitant processes in white adipose tissue expansion: hyperplasia (increased numbers of fat cells associated with the differentiation of adipocyte precursors) and hypertrophy (increased size of fat cells) [150–152]. Several studies have shown a close relationship between ROS and fat mass expansion [153, 154]. Fat accumulation parallels with ROS, as demonstrated by an increase of ROS accumulation during adipocyte 3T3-L1 alteration [155, 156]. Leptin, a white adipose tissue-derived hormone, has been reported to promote the elevation of ROS accumulation in endothelial cells [157, 158]. NF-*κ*B can be stimulated by leptin in an oxidant-dependent manner. This finding is linked with an increased expression of monocyte chemoattractant protein-1 (MCP-1), which enhances atherosclerosis by supporting the relocation of inflammatory cells [152, 159]. Further, leptin also activates ROS in vascular smooth muscle cells via the protein kinase C-dependent activation of NAD(P)H oxidase [160]. Leptin promotes the release of active macrophage lipoprotein lipase via an oxidative stress-dependent pathway, signifying a proatherogenic effect of leptin on macrophages in diabetes [157]. By contrast, the exposition of adipocytes to high ROS levels suppresses the secretion and expression of adiponectin [161], an adipokine that shows anti-inflammatory, antiatherogenic, and insulin-sensitizing properties [162]. Collectively, systemic oxidative stress-associated HFD and obesity may lead to insulin sensitivity of metabolic organs, which thus promotes the inflammatory response [163].

Obesity has been demonstrated to drive the development of insulin resistance. However, not all obese individuals develop type 2 diabetes mellitus or insulin resistance, indicating that the biological mechanism underlying the association between obesity and insulin resistance must be well-controlled under certain circumstances [164]. Obesity has become an epidemic and represents the major risk factor for several chronic diseases, including diabetes, CVD, and cancer [165]. Therefore, the present study focused on the detrimental impact of oxidative stress on diabetes, CVD, and cancer outcomes.

### 4.2. Diabetes

Type 2 diabetes is the most common metabolic disorder, affecting 422 million people worldwide in 2014 [166], with nearly half of all deaths attributable to high blood glucose [166]. Type 2 diabetes is currently the most common form of the disease, representing nearly 90–95% of diabetes mellitus cases. Diabetes mellitus is a complex and progressive disease that is accompanied by several complications such as nephropathy, retinopathy, neuropathy, and micro- and macrovascular damage [167].

Oxidative stress has been identified as a major risk factor in the development of diabetes [168]. Numerous risk factors including increased age, unhealthy dietary intake, and obesity all lead to an oxidative environment that may modify insulin sensitivity either via the elevation of insulin resistance or the impairment of glucose tolerance [169]. The mechanisms that implicate these diseases are complex and involve several cell signaling pathways [170]. Hyperglycemia is linked to diabetes and subsequently contributes to its progression and an overall oxidative environment [171]. Macro- and microvascular complications contribute to the morbidity and mortality of diabetic patients, and all these factors are associated with oxidative stress [172].

The derangement of molecular and cellular processes is common in type 2 diabetes, particularly in *β* cells. Pathophysiologically, ROS and RNS, such as H_2_O_2_, superoxide anion (O_2_^∙−^), NO, peroxynitrite (ONOO−), and hydroxyl radical (OH^∙^), all contribute to primary physiologic and metabolic processes. Mitochondrial function impairment leads to a reduction in ATP generation capacity, which in turn leads to *β* cell glucose-stimulated insulin secretion (GSIS), the NADPH complex, and Ca^2+^ signaling related to neurotransmission [173, 174].

Insulin resistance plays a predominant role in the development and progression of metabolic dysfunction associated with obesity. Insulin resistance refers to the impairment of the cellular response in insulin-sensitive tissues such as skeletal muscle, adipose, liver, and brain tissues [175–177]. Subsequently, this may lead to a reduction of glucose uptake, accompanied by the elevation of hepatic glucose output, and thereby contribute to plasma glucose concentrations [178]. The subsequent changes of glucose homeostasis may place a burden on pancreatic *β* cells to secrete and produce more insulin to restore normal blood carbohydrate levels [179]. Nonetheless, this compensatory mechanism may ameliorate glucose levels in an early or prediabetes condition, characterized by continuous insulin resistance and high exposure of *β* cells to blood glucose and lipids [180]. This may boost *β* cell failure and dysfunction and culminate in overt diabetes [176].

Pancreatic islets are highly vascularized and specialized structures that control the nutrient contents in the bloodstream and are mainly comprised of five cell types: *α* cells, *β* cells, *δ* cells, ghrelin cells (*γ* cells), and pancreatic peptide- (PP-) secreting cells [181]. Islets generate blood from the splenic branches and pancreaticoduodenal arteries and interact to increase dietary nutrients to secrete insulin from *α* and *β* cells into glucagon and the bloodstream, respectively (during nutrient-deprived conditions such as starvation and fasting) [175]. The pancreatic *β* cell response to glucose depends on the acute regulation of intracellular or extracellular ROS and RNS [173, 174]. The elevation of glycolytic flux promotes ATP production and oxidative phosphorylation, which subsequently results in the formation of O_2_^∙−^ released from the electron transport chain [182]. Additionally, an initial adaptive response is modulated through the pentose phosphate pathway in which surfeit glucose is converted to pentose and glucose carbon is deviated away from excessive oxidative and glycolysis phosphorylation. However, shuttling glucose in this direction may also increase NADPH oxidase (NOX) activity and subsequently lead to increased O_2_^∙−^ synthesis. Indeed, high glucose levels may increase ROS through other possible mechanisms, such as the generation of advanced glycation end products (AGEs) and glucose autoxidation [183].

Once insulin is released into the blood circulation by *β* cells in response to increased blood glucose levels, insulin exhibits its anabolic effects through the transmembrane insulin receptor (IR) in target tissues. Interaction with insulin fosters the autophosphorylation of the receptor with the phosphorylation and recruitment of insulin receptor substrate (IRS) proteins and the stimulation of other related downstream signaling cascades, such as protein kinase B (Akt) and phosphatidylinositol-3-kinase (PI3K) [184]. Akt has been identified as a primary regulator in vesicle translocation of glucose transporter type 4 (GLUT-4) to the plasma membrane, which is crucial in the intracellular uptake of free glucose in insulin-sensitive tissues [48].

Numerous studies have indicated that there is an association between increased nitrosylation and carbonylation of proteins in obese- or insulin-resistant phenotypes and insulin-sensitive tissues [110, 185–187]. This suggests that an insulin-resistant phenotype may promote the reduction of insulin receptor expression. Thus, prolonged hyperinsulinaemia and chronic hyperglycemia, along with increased ROS and RNS levels, are hypothesized to influence insulin receptor gene expression through the derangement of key transcription factors such as high mobility group AT-hook 1 (HMGA-1) [188]; they may also increase insulin receptor-desensitization, which under normal circumstances is a process under the negative-feedback control [189, 190]. Taken together, the development and progression of diabetes mellitus is associated with *β* cell dysfunction and insulin resistance, and this phenomenon is normally related to obesity [175].

### 4.3. Cardiovascular Disease

Oxidative stress is implicated in the progression and development of cardiovascular disease (CVD) [191]. Its burden is attributable to lifestyle factors, particularly smoking, alcohol consumption, sedentary lifestyles, and dietary intake [192]. In Malaysia, western dietary habits that are high in fat and low in dietary fiber lead to the increase in CVD incidence [193]. Chronic and low-grade inflammation has been suggested as a major pathophysiology in obesity and its associated diseases such as CVD [194]. C-reactive protein (CRP) has been shown to be an independent risk factor for the development of CVD [195, 196]. The elevation of CRP in obesity could be attributed to macrophage infiltration into the expanded adipose tissue and subsequently leads to the production and release of macrophage-derived proinflammatory cytokines such as IL-6 and TNF-*α* [197, 198].

One common feature of CVD is increased oxidative stress in the heart [199]. Specifically, systemic oxidative damage in patients with CVD was due to ROS accumulation and reduced antioxidant defense [200]. A HFD increased ROS accumulation and reduced antioxidant capacity, thus causing a variety of disorders including endothelial dysfunction, which is characterized by a decreased bioavailability of vasodilators, namely, NO, and promotes endothelium-derived contractile factors causing atherosclerotic disease [201]. One potential biological mechanism linking cardiac oxidative stress has been described by Ilkun and Boudina [201] and includes mitochondrial dysfunction, increased fatty acid oxidation, and increased NADPH oxidase activity. Ilkun and Boudina [201] demonstrated that the modes of action underlying cardiac pathology are complex and might include altered calcium homeostasis, lipid accumulation, abnormal autophagy, increased fibrosis and stiffness, increased oxidative stress, and mitochondrial dysfunction. Collectively, mitochondrial and extramitochondrial sources of ROS and a reduction of antioxidant defense mechanisms have occurred in the myocardium of human and animals [201].

### 4.4. Cancer

Research has demonstrated that high oxidative stress leads to cancer, including colorectal cancer [202]. Oxidative stress is hypothesized to be associated with obesity and cancer. A study in an animal obese model of nonalcoholic steatohepatitis supports these hypotheses, suggesting that the absence of adiponectin promotes hepatic tumor formation and elevates oxidative stress [203]. Indeed, ROS plays a crucial role in cancer development [204, 205]. The elevation of ROS leads to increased mutation rates or susceptibility to mutagenic agents and thus contributes to DNA damage during the early stages of carcinogenesis [205]. The elevation of ROS has also been demonstrated in tumor proliferation via the ligand-independent transactivation of receptor tyrosinekinase [204], which can promote metastasis and the invasion of cancer cells [206]. Semenza [207] observed that ROS can promote the stabilization of hypoxia-inducible factor 1, a transcription factor of vascular endothelial growth factor, which facilitates tumor angiogenesis.

Intriguingly, data from a previous study have shown that insulin is a proliferation factor for prostate cancer; thus, the reduction of carbohydrates may subsequently decrease serum insulin and slow down prostate cancer proliferation [208]. Epidemiological studies have shown that patients with type 2 diabetes and obesity have a greater likelihood of having liver, colorectal, breast, and pancreatic cancers [209, 210]. These findings suggest that leptin [203, 211], insulin/insulin-like growth factor-1 [212, 213], adiponectin [203, 211], and inflammation [214, 215] are additive between type 2 diabetes or obesity and cancers. Fat accumulation is often linked with systemic oxidative stress via elevation of ROS [114]. A previous study stated that increased oxidative stress can lead to chronic inflammation, which in turn could modulate chronic diseases such as cancer [216]. Oxidative stress can trigger a wide range of transcription factors such as Wnt/*β*-catenin, NF-*κ*B, and nuclear factor E2-related factor 2 (*Nrf2*) and thereby activates inflammatory pathways [216]. Taken together, these findings suggest that increased circulating or local ROS levels derived from the expansion of the adipose tissue in a tumor environment provoke oxidative stress within tumor cells and thereby lead to an increased risk for cancer progression in patients with type 2 diabetes or obesity.

## 5. Diet Ameliorates Oxidative Stress-Induced Diseases

**Figure 1:**
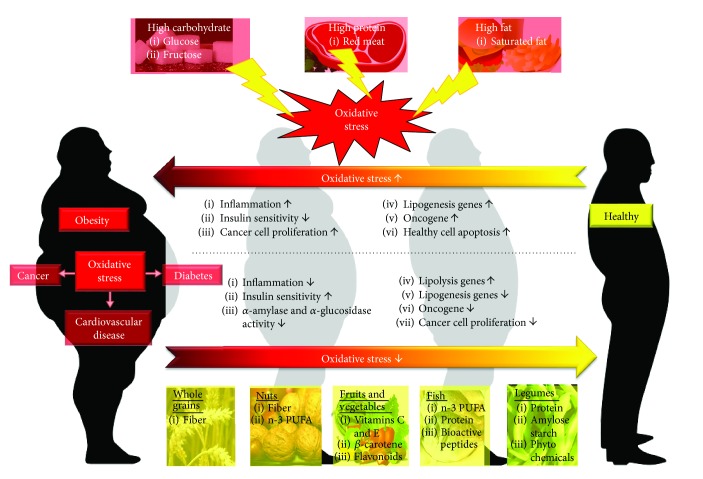
Dietary intake patterns affect human health state. High-carbohydrate and an animal-based protein diet and excessive fat consumption will eventually lead to obesity as well as other obesity-related diseases such as cardiovascular diseases (CVD), diabetes, and cancer. The key pathway involved in the pathogenesis is via the elevation of oxidative stress. Subsequently, inflammation occurs resulting in the reduction of insulin sensitivity, increased cancer cell proliferation, involvement of gene in lipogenesis, and cancer development of which is activated and accompanied by apoptosis of healthy cells. To revert these unhealthy conditions, consumption of healthy diet is essential. Healthy diet includes whole grains, nuts, fruits and vegetables, fish, and legumes. In general, a healthy diet contains dietary fiber, unsaturated fatty acids like monounsaturated fatty acid (MUFA) and n-3 polyunsaturated fatty acid (n-3 PUFA), protein, vitamins, minerals, and others health-promoting components. All these components exhibit antioxidant ability thereby reduce oxidative stress. The healthy diet could reduce inflammation, cancer development, and lipogenesis transcriptional expression. It also increases insulin sensitivity accompanied by the reduction of *α*-amylase and *α*-glucosidase activity. A healthy dietary pattern is crucial for maintaining good health.

Oxidative stress is increased in diabetic patients and cancer cells [217, 218]. Higher intracellular glucose concentrations can generate ROS via several pathways [219], and the progression and development of these diseases could be prevented by changing dietary habits [220]. It was evident that high-glucose and an animal-based protein diet and excessive fat consumption can promote oxidative stress [221], for example, excessive omega-6 stimulates inflammation [222]; however, there are other dietary choices (the Mediterranean and Okinawan diets of the Greek and Japanese populations) that can reduce inflammation [223].  Figure 1 summarizes the dietary intake pattern in relation to human health.

### 5.1. Whole Grains

Numerous components of the diet may promote inflammation. Whole grains comprised of germ, endosperm, and bran are rich in vitamins, fibers, minerals, and phytochemicals such as carotenoids, lignans, vitamin E, inulin, *β*-glucan, sterols, and resistant starch [224]. As an example, the fiber found in whole grain foods appears to play a role in immune-modulating functions [225, 226]. Fiber affects microbiota in the gut [227], which affects immune function [228]. In support of this, the intake of whole grains such as sorghum benefits the gut microbiota and indices associated with oxidative stress, obesity, inflammation, hypertension, and cancer [229]. Whole grain foods are rich in phytochemicals and provide protection against oxidative stress, which can result in inflammation. Polyphenol compounds present in wheat sprouts may benefit a certain group of the population because they appear to combat oxidative stress associated with obesity [230] and enhance glucose metabolism [231].

Data from a meta-analysis have demonstrated that a high intake of whole grain products is associated with a reduction of total cancer risk [232]. In a Scandinavian HELGA cohort study, intakes of whole grains were found to be inversely associated with colorectal cancer incidence [233]. A study by Tan et al. [202] and Tan et al. [234] further supported the role of a unique complex of bioactive constituents in brewers' rice, which is a rice by-product in the rice industry that exerts significant nutritional value to combat colon carcinogenesis. Anti-inflammatory effects of brewers' rice protect against oxidative stress and free radical damage by improved antioxidant enzymes such as MDA, SOD, and NO. They also inhibit DNA damage caused by ROS via the upregulation of the *Nrf2* signaling pathway. Several studies have also reached a similar finding, in which rice by-products have an antiproliferative activity against cancer [235, 236]. Strikingly, feeding with brewers' rice not only reduced the number of aberrant crypt foci (ACF) [237]; in fact, the relative proportions of natural antioxidant compounds in brewers' rice have also been reported to attenuate liver and kidney damage in azoxymethane-induced oxidative stress in rats, as reported by Tan et al. [238], suggesting that bioactive constituents present in whole grains may ameliorate oxidative stress.

In addition to the effects observed on cancer, germinated brown rice has been extensively studied in the past few decades. Germinated brown rice has a significant nutritional value. In addition to containing high amounts of minerals, vitamins, and fiber, germinated brown rice is also rich in a variety of bioactive compounds and has drawn a great deal of interest in the prevention of CVD risk. These bioactive compounds were demonstrated to have antioxidant activities that are suggested to alleviate CVD risk via the modulation of hepatic cholesterol metabolism and oxidative stress [239, 240]. Accordingly, germinated brown rice modulates lipid metabolism via the transcriptional regulation of peroxisome proliferator-activated receptor gamma (PPAR*γ*), hepatic lipoprotein lipase (LPL), ATP-binding cassette, subfamily A (ABCA), v-akt murine thymoma viral oncogene homologue 1 and homologue 3 (AKT1 and AKT3), and adiponectin [239]. In this regard, natural components present in whole grain such as polyphenolic compounds have the potential to suppress proinflammatory immune signaling and subsequently improve lipid metabolism and inhibit cancer development [241].

Notably, the nutritional values of fiber components such as arabinoxylans and *β*-glucans are also found in whole grains. Studies have revealed a positive association between wheat and rye arabinoxylans and water-soluble maize on caecal fermentation, the reduction of serum cholesterol, and the production of short-chain fatty acids [242, 243]. Dietary fibers present in whole grain also play a central role to enhance immune function through the production of short-chain fatty acids, suggesting that increasing the intake of fermentable dietary fiber may be vitally important in reducing inflammation [244, 245]. Short-chain fatty acids may promote T helper cells, neutrophils, macrophages, and cytotoxic activity in natural killer cells [246]. Further, the fermentation of dietary fiber in the colon and changes in gut microbiota are associated with impaired gastrointestinal tolerance [247]. Together with the gut immune system, mucosal and colonic microflora prevent pathogenic bacteria from invading the gastrointestinal tract [248]. The intestinal flora salvages energy via the fermentation of undigested carbohydrates in the upper gut [246]. The predominant substrates are dietary carbohydrates and mucus, which escape digestion in the upper gastrointestinal tract [246]. These include nonstarch polysaccharides (such as hemicelluloses, celluloses, gums, and pectins), resistant starch, sugar alcohols, and nondigestible oligosaccharides [246]. The primary fermentation pathway produces pyruvate from hexoses in undigested carbohydrates [246]. Colonic bacteria use a wide range of carbohydrates to hydrolyze enzymes and produce methane, hydrogen, short-chain fatty acids (primarily butyrate, propionate, and acetate), carbon dioxide, and lactate [249]. In this regard, these components activate fermentation, increase bacterial and fecal mass, and ultimately lead to a stool bulking effect [246]. Overall, this suggests that the protective effect of whole grains on oxidative stress may be mediated partly via the synergistic/additive effects of these bioactive components.

### 5.2. Nuts

When a landmark epidemiological study found that a high frequency of nut consumption was related to a reduction of CVD [250], nuts were brought from obscurity to prominence as a crucial health food. In the last 15 years since this first epidemiological study, scientific research on the health effects of nuts has not only focused on the area of coronary heart disease and its risk factors but has also extended to other areas of health. In addition, clinical trials have found that diets enriched with nuts reduce oxidative stress and inflammation [251] and alleviate endothelial dysfunction or insulin resistance [252]. Another clinical study consistently reported a hypocholesterolemic potential of regular nut consumption, which partly explains how walnuts reduce the risk of CVD [253].

Nuts are not only a high-fat and energy-dense food but they are also rich in bioactive constituents [254] that are believed to have anti-inflammatory and anticarcinogenic properties including folic acids and several phytochemicals [255, 256]. Notably, collective findings suggest that a protective role of nuts on colorectal and endometrial cancer prevention is possible [257–259].

A crucial underlying mechanism of action that has been proposed to explain an inverse relationship between the frequency of nut-enriched consumption and risk of obesity is unsaturated fatty acids. Healthy fats (unsaturated fatty acids) in nuts contribute to the prevention of diabetes and CVD risk. By contrast, nuts are complex food matrices that are also a source of other bioactive constituents, namely, tocopherols and phenolic compounds [260]. Compelling evidence suggests that monounsaturated fatty acids (MUFAs) and polyunsaturated fatty acids (PUFAs) are more readily oxidized [261] and have a greater thermogenic effect [262] than do saturated fatty acids, which might contribute to less fat accumulation. Due to their unique fat and nonfat composition, nuts are more likely to mediate inflammation and oxidative stress.

Because nuts contain the abundance of unsaturated fatty acids, protein, and fiber, they are a highly satiating food [263]. Thereby, after consuming nuts, hunger is reduced and subsequent food intake is curtailed [264]. The physical structure of nuts may also lead to their satiety effect because they must be masticated, small enough for swallowing. Mastication stimulates nutrient, mechanical, and sensory signaling systems that may alter appetitive sensations [265]. Additionally, a small degree of fat absorption may occur after nut consumption because fat is found within the wall cellular structures that are not fully digested in the gut [266], which could be compounded by incomplete mastication [267]. Data from population-based studies indicate an inverse relationship between nut intake, such as almonds and CRP [268, 269]. Plasma IL-6 levels were reduced after a Mediterranean diet with nuts compared to a control diet [270, 271]. Similarly, previous studies reported by Zhao et al. [272] and Zhao et al. [273] also evaluated walnuts rich in PUFAs and, in particular, alpha-linolenic acid (ALA), in relation to proinflammatory cytokine production [273] and inflammatory markers [272] by blood mononuclear cells. The data showed that compared to the average American diet, the CRP levels were reduced by 75% in subjects consuming an ALA diet; conversely, levels in subjects consuming the linoleic acid (LA) diet decreased by 45% [272]. Indeed, reductions in multiple inflammatory markers such as IL-6, IL-1*β*, and TNF-*α* produced by cultured mononuclear cells were observed from subjects who consumed an ALA-enriched diet [273].

Based on the findings for marine-derived omega-3 PUFA, ALA would be expected to have anti-inflammatory properties. This was evaluated in a clinical study with a relatively small observed effect [274]. However, an *in vitro* study in which THP-1 cells were supplemented with LA, ALA, docosahexaenoic acid (DHA), and palmitic acid in the presence of lipopolysaccharide [275] showed a significant reduction in TNF-*α*, IL-1*β*, and IL-6 after treatment with DHA, ALA, and LA compared to palmitic acid, indicating that ALA present in walnuts elicits an anti-inflammatory response. Notably, cellular adhesion molecules are biochemical markers of endothelial dysfunction concomitantly with inflammation. In a further study focused on CVD outcomes, Zhao et al. [273] compared hypercholesterolemic subjects who consumed a diet high in ALA, a diet high in LA, and an American diet, respectively. The data showed that participants who consumed 15 g of walnut oil along with 37 g of walnuts/daily for 6 weeks demonstrated a reduction in CRP, cellular adhesion molecule soluble intercellular adhesion molecule (sICAM) 1, and E-selectin. Importantly, some research has emerged to suggest that CVD risk factors negatively affect endothelial function and are involved in the modulation of LDL cholesterol [276, 277]. In support of this, the acute consumption of walnuts oils is favorably affected and shows a better endothelial function [278]. Further, walnuts and walnut oil may influence inflammation, at least in part, via the elevation of cholesterol efflux, which is a reverse cholesterol transport that is crucial for the removal of cholesterol from peripheral tissues and indicates cardioprotective effects [253]. Taken together, nuts seem a good dietary choice for providing nutrients and preventing obesity and other chronic diseases. However, the bioactive components responsible for the effects that we stated above require further elucidation.

### 5.3. Fruits and Vegetables

Fruits and vegetables are rich in minerals, vitamins, and dietary fiber. High intakes of fruits and vegetables are inversely associated with mortality and the incidence of obesity-related diseases such as CVD, type 2 diabetes, and cancer [279]. Such protection has been accredited to antioxidant vitamins such as *β*-carotene, vitamin E, and vitamin C [280]. In general, more than 85% of the total antioxidants in fruits and vegetables are hydrophilic antioxidants [281]. Beta-carotene and vitamins E and C are vitally important for the proper regulation of physiological function [282]. The essential role of vitamin E in maintaining the oxidative-antioxidant balance is well-recognized, yet vitamin C can enhance the antioxidant protection [282]. Beta-carotene is usually found in bright-colored fruits and vegetables [283]. It has been demonstrated to maintain the immune system and exert an ability to decrease LDL-cholesterol oxidation through the modulation of antioxidant enzymes [283]. In addition to the vitamin antioxidants stated above, other dietary components such as flavonoids may protect against oxidative stress. Flavonoids are plant polyphenolic compounds ubiquitous in fruits and vegetables. Flavonoids exert multiple biological activities such as antitumor effects, anti-inflammatory activity, antioxidant activity, and antimicrobial action, and they suppress platelet aggregation [284].

An animal study has demonstrated that a diet supplemented with *β*-carotene from fruit significantly downregulated the expression of fatty acid synthase, acetyl-CoA carboxylase, and fat synthesis-related genes [285]. Findings from a population-based study mirror some of those from preclinical data obtained from an *in vivo* study. Data from a population-based study reported that high intakes of fruits and vegetables significantly decreased energy consumption, waist circumference, body weight, and sagittal abdominal diameter in overweight and obese men and women [286].

Compelling epidemiological studies have revealed that intakes of fruits and vegetables induce protective cardiometabolic effects. A study showed that encapsulated fruit and vegetable-concentrated juice decreased total cholesterol, LDL-cholesterol, plasma TNF-*α*, and systolic blood pressure, in addition to increasing total lean mass [287]. The improvements in these indices could be attributed to the alteration of gene expression via several signaling pathways such as AMP-activated protein kinase (AMPK) and NF-*κ*B associated genes [287]. Body composition, blood lipids, and systemic inflammation were improved in obese subjects after consuming fruits and vegetables and thus provide a useful approach for reducing the obesity-induced chronic diseases risk [288]. Further, fruits and vegetables can also prevent CVDs or assist with the restoration of function and morphology of vessels and the heart after injury. Fruits and vegetables are thought to protect against CVD by regulating lipid metabolism, protecting vascular endothelial function, suppressing platelet function, modulating blood pressure, inhibiting thrombosis, attenuating inflammation, alleviating ischemia/reperfusion injury, and reducing oxidative stress [289, 290].

In addition to the effects observed in obesity and CVD, a beneficial effect of fruit and vegetable consumption in human has also been reported on the incidence of type 2 diabetes. Data from a meta-analysis included a study from 1966 to 2014 that demonstrated that a high intake of fruit, particularly berries, and yellow, cruciferous, green leafy vegetables or their fibers, is negatively linked to type 2 diabetes [291]. As an example, lactucaxanthin (Lxn), a carotenoid in lettuce (*Lactuca sativa*), suppresses *α*-amylase and *α*-glucosidase activity both *in vitro* and in diabetic rats [292]. Such findings highlight the role of unique complexes of bioactive components in fruits and vegetables.

Fruits and vegetables not only reduce obesity, CVD, and diabetes but they also inhibit several cancers, demonstrating the numerous functional potentials of fruits and vegetables. Epidemiological studies have shown an inverse relationship between fruit and vegetable intakes and cancer risks such as colon, breast, and prostate cancers. This suppressive effect was mainly observed in cruciferous and green-yellow vegetables [293] via the modulation of genes involved in proliferation and glucose metabolism and the induction of several antioxidant genes [294]. Notably, dietary fiber in fruits and vegetables will undergo fermentation by gut microbiota, which may lead to the production of short-chain fatty acids. Short-chain fatty acids such as acetate [295], butyrate [296], and propionic acids [297] may have protective effects against cancers. It is possible that only certain types of fruits and vegetables confer protection against oxidative stress [283]. Since some bioactive compounds regulate the same gene expression and pathways targeted by drugs, diets high in fruits and vegetables in combination with medical therapies are being considered as a novel treatment strategy [298]. Overall, bioactive constituents in fruits and vegetables might be promising tools for the alleviation of a wide range of diseases [299].

### 5.4. Fish

Fish is an essential source of dietary protein, omega-3 fatty acids, and minerals. Nakamura et al. [300] demonstrated that individuals who consume fish daily were inversely associated with obesity compared to those with normal weight or underweight. The intake of fish has been linked to a reduced risk of obesity [301], yet the composition of fish often includes representative PUFA amounts, such as n-3 fatty acids, whose chemical structure makes them prone to peroxidation and are found abundantly in fatty fish. Therefore, our body becomes more susceptible to oxidative stress and subsequently activates the lipid peroxidation process [302]. Undoubtedly, PUFA intake is essential as they have well-established health benefits especially in preventing heart disease [303]. However, it is recommended to have an adequate vitamin E to match the increased of PUFA intake [304]. This is because lipophilic antioxidant vitamin E plays a vital role in protecting PUFA [305]. In addition, an animal study has shown that the vitamin E requirement is increased almost proportionally with the degree of unsaturation of the PUFA [304].

The consumption of lean fish has a beneficial impact on insulin sensitivity, glucose homeostasis, and lipid metabolism [306, 307]. Aadland et al. [306] further demonstrated that intakes of lean fish for 4 weeks reduced the ratio of total to HDL cholesterol in serum, decreased the VLDL concentration, and reduced fasting and postprandial triacylglycerol (TAG) compared to those with a nonseafood diet, suggesting the cardioprotective potential of lean-seafood intake. A similar dietary intake was also found to reduce the urinary excretion of metabolites involved in mitochondrial lipid and energy metabolism, possibly facilitating a higher lipid catabolism [308]. Intriguingly, lean fish contains relatively low amounts of marine n-3 fatty acids, and thereby the beneficial effects of fish are not solely ascribed to the lipid composition.

Dietary protein has been suggested as the most effective food macronutrient to provide a satiating effect. Therefore, protein-rich foods can facilitate in the modulation of food intake, promoting body weight loss and maintaining body weight thereafter. Glucagon-like peptide-1 (GLP-1) release stimulated by a high-protein meal is evoked by carbohydrate content. Indeed, cholecystokinin (CCK) and peptide YY (PYY) release is activated by a high-protein meal [309].

Fish not only contains macronutrients but also has a substantial antioxidant source due to its composition and offers a relatively low level of saturated fat compared to other food items. Taurine, an amino acid that is abundantly found in fish, is a vital antioxidant source. Studies have shown that taurine can effectively combat metabolic syndrome by regulating glucose metabolism, reducing triglycerides to prevent obesity, regulating the renin-angiotensin-aldosterone and kallikrein-kinin systems to decrease blood pressure, and lowering cholesterol (particularly reducing VLDL + LDL cholesterol and promoting HDL cholesterol) to prevent diet-induced hypercholesterolemia [310].

Notably, the production of fish protein peptides (hydrolysates) maximizes the usage of fish protein because peptides have a health-promoting potential [311]. Techniques such as autolysis, thermal hydrolysis, and enzymatic hydrolysis have been developed to produce fish hydrolysates. The antiviral-, cardioprotective- (antihypertensive, antiatherosclerotic, and anticoagulant), analgesic-, antimicrobial-, antioxidative-, antitumor-, immunomodulatory-, neuroprotective, and appetite-suppressing activities have drawn attention from the pharmaceutical industry, which attempts to design the treatment and prevention of certain diseases [312]. Lassoued et al. [313] and Razali et al. [314] reported that peptides derived from fish proteins exhibit significant antioxidative activity in oxidative systems. The dietary intake of antioxidant compounds can strengthen the body's oxidant status and facilitate a balanced condition in terms of oxidants/antioxidants in the body.

In addition to fish and its protein peptides, neovastat (AE-941), a liquid extract derived from the cartilage of sharks, exerts antiangiogenic, anti-inflammatory, and antitumor properties both *in vitro* and *in vivo* [315]. These favorable effects are mediated via the suppression of matrix metalloproteinases (MMP)-2, MMP-9, and MMP-12 and the activation of tissue plasminogen activator enzymatic activities [315].

Another metabolic disorder is hypertension, which occurs when renin produces *angiotensin I* from *angiotensinogen*. The angiotensin I-converting enzyme (ACE) cleaves *angiotensin I* to *angiotensin II*, which is a potent vasoconstrictor [316]. Accordingly, Balti et al. [317] have sourced bioactive constituents from different types of fish in ACE-inhibitor activity studies with molecular weights of <10 kDa. From a review study, Balti et al. [317] found that bioactive peptides are suitable competitive inhibitors that can bind to the active site of ACE and thereby block its activity. Collectively, it remains unknown whether PUFA content or its antioxidant is responsible for its beneficial effects; thus, further study is necessary to conclusively resolve the question behind the anti-inflammatory effects of fish.

### 5.5. Legumes

Legumes are a primary component of the Mediterranean diet. They are rich in fiber and protein, which can facilitate in lowering energy density and reducing the glycemic response [318]. Legumes also contain B vitamins and minerals, such as potassium, calcium, and iron. Most of the nutritional value in legumes is contributed by their relative proportions of protein, fibers [319], and phytochemicals such as isoflavones, phytoestrogens, saponins, oligosaccharides, lectins, and phenolic compounds [320]. Due to their high nutritional values, legume intake has been demonstrated to have beneficial effects in the prevention of obesity and other related disorders [321].

Compared to those who rarely or never consume legumes, adults who consume legumes have a significantly lower body mass index and waist circumference. Children who consume legumes had smaller waist circumferences compared to those who never consume legumes [322]. Shinohara et al. [323] further demonstrated that ethanol extracts of chickpeas improved total lipid indices and gene expression associated with fatty acid metabolism in adipocytes. Studies have shown that enzymes involved in lipogenesis such as AMPK, acetyl-CoA carboxylase (ACC), and liver kinase B1 (LKB1) were inactivated by phosphorylation. Further, lipolysis was increased by the extract through the stimulation of palmitoyltransferase 1 (CPT1) and uncoupling protein 2 (UCP2), which has been reported as a crucial protein in fatty acid oxidation [323].

Starch digestibility and composition influence glycemic response. Legumes are high in amylose starch. Nonetheless, the digestion of high amylose starch is significantly lower compared to that of high amylopectin starch [324]. Yang et al. [325] reported a more sustainable plasma glucose level after a high-amylose meal compared to a high-amylopectin meal [325]. Furthermore, legumes have a high protein content; thus, the interaction of protein-starch may further hamper digestibility [326]. Moreover, high amounts of dietary fiber markedly reduced the extent and rate of legume starch digestibility. A high intake of fiber may promote satiety, enhance insulin resistance, and decrease the glycemic response [327]. Evidence from epidemiological studies shows that legume intake is negatively associated with fasting glucose levels [328].

Notably, data from large-scale epidemiological studies found that legume consumption is negatively associated with CVD mortality. Compared to the highest and lowest legume consumption, high legume consumption showed a 6% decreased risk of CVD [329]. Isoflavones are believed to have hypolipidemic activity by binding with estrogen receptors when circulating estrogen is low and thereby translocating to the nucleus, which interacts with a DNA sequence near the promoter region of target genes and results in DNA transcription [330]. Through this mechanism, isoflavone may act as a ligand for lipid-regulating proteins including PPAR, farnesoid X receptor, and liver X receptor, which facilitates cholesterol reabsorption, bile acid synthesis, and hepatic lipid synthesis [331].

In addition to the effects mentioned above, legumes have the potential to protect against cancers. For example, soy food protects against estrogen receptor-negative breast cancer [332]. A study reported by Guo et al. [332] demonstrated that in women with high soy intakes, tumor suppressor genes were upregulated (miR-29a-3p and IGF1R), and oncogenes were downregulated (KRAS and FGFR4). Consistent with the study reported by Guo et al. [332], green pea- (*Pisum sativum*) extracted lectin has also been reported to have antiproliferative activity against liver cancer cell lines [333]. Despite the limited available evidence to draw a firm conclusion, some studies suggest that legumes may be potentially beneficial to some population segments. Collectively, future studies may elucidate the role of legumes in human health, yet their use within a balanced diet should be considered in the absence of clear contraindications.

## 6. Summary and Future Prospects

This review has provided clear evidence of the identification of known sources of nutritionally mediated oxidative stress as a mediating pathway for both risks of obesity and other obesity-associated diseases. Oxidative stress is a central player of metabolic ailments associated with high-carbohydrate and animal-based protein diets and excessive fat consumption. There is inconsistent research supporting the clinical use of antioxidant agents in preventing or delaying the onset and progression of metabolic disorders such as diabetic complications and cancer [334–337], and most clinical studies are limited in their sample size and duration of the study. Despite this, preclinical studies *in vitro* and animal experiments have provided in-depth insight into the modulation of these diseases. Several anti-inflammatory dietary sources such as whole grains, nuts, fruits and vegetables, and others can delay the onset of insulin resistance, prevent adipocyte and endothelial dysfunctions, and prevent tumor proliferation by reacting with oxidizing free radicals and inhibiting the inflammatory response. Therefore, more randomized clinical trials are warranted to evaluate the overall long-term effects of dietary intervention.

## 7. Conclusions

The available research strongly supports that a diet high in carbohydrates and animal proteins and excessive fat consumption produces ROS and subsequently leads to oxidative stress. The best dietary advice for the prevention and management of obesity and other metabolic disorders includes replacing refined carbohydrates with whole grains, increasing fruits and vegetables, substituting total and saturated fat with MUFAs, and consuming a moderate amount of calories with an ultimate goal of maintaining an ideal body weight. Overall, further studies are warranted to gain a better understanding of the types and the degree of ROS generation in relation to diet-induced metabolic disorders.
